# The Role of Nuclear Medicine Imaging with ^18^F-FDG PET/CT, Combined ^111^In-WBC/^99m^Tc-Nanocoll, and ^99m^Tc-HDP SPECT/CT in the Evaluation of Patients with Chronic Problems after TKA or THA in a Prospective Study

**DOI:** 10.3390/diagnostics12030681

**Published:** 2022-03-10

**Authors:** Ramune Aleksyniene, Victor Iyer, Henrik Christian Bertelsen, Majbritt Frost Nilsson, Vesal Khalid, Henrik Carl Schønheyder, Lone Heimann Larsen, Poul Torben Nielsen, Andreas Kappel, Trine Rolighed Thomsen, Jan Lorenzen, Iben Ørsted, Ole Simonsen, Peter Lüttge Jordal, Sten Rasmussen

**Affiliations:** 1 Department of Nuclear Medicine, Aalborg University Hospital, 9000 Aalborg, Denmark; victor.iyer@akademiska.se (V.I.); hcb@rn.dk (H.C.B.); mf@rn.dk (M.F.N.); 2 Department of Radiology and Molecular Medicine, University Hospital Uppsala, 75237 Uppsala, Sweden; 3 Department of Clinical Medicine, Aalborg University, 9220 Aalborg, Denmark; vekh@rn.dk (V.K.); sten.rasmussen@rn.dk (S.R.); 4 Orthopaedic Research Unit, Aalborg University Hospital, 9000 Aalborg, Denmark; 5 Department of Clinical Microbiology, Aalborg University Hospital, 9000 Aalborg, Denmark; hcs@rn.dk (H.C.S.); lone_heimann@hotmail.com (L.H.L.); 6 Interdisciplinary Orthopaedics, Department of Orthopaedic Surgery, Aalborg University Hospital, 9000 Aalborg, Denmark; ptn@rn.dk (P.T.N.); andreas.kappel@rn.dk (A.K.); on.os@nja.dk (O.S.); 7 Center for Microbial Communities, Aalborg University, 9220 Aalborg, Denmark; trt@bio.aau.dk; 8 Danish Technology Institute, Medical Biotechnology, 8000 Aarhus, Denmark; jnl@dti.dk (J.L.); plj@sbtinstruments.com (P.L.J.); 9 Department of Infectious Diseases, Aalborg University Hospital, 9000 Aalborg, Denmark; iben.oersted@rn.dk

**Keywords:** hybrid imaging, nuclear imaging, FDG PET/CT, labeled leucocyte imaging, dual-isotope WBC/bone marrow scan, bone scan, prosthetic joint infection, periprosthetic infection

## Abstract

Background: The aim of this prospective study was to assess the diagnostic value of nuclear imaging with ^18^F-FDG PET/CT (FDG PET/CT), combined ^111^In-WBC/^99m^Tc-Nanocoll, and ^99m^Tc-HDP SPECT/CT (dual-isotope WBC/bone marrow scan) for patients with chronic problems related to knee or hip prostheses (TKA or THA) scheduled by a structured multidisciplinary algorithm. Materials and Methods: Fifty-five patients underwent imaging with ^99m^Tc–HDP SPECT/CT (bone scan), dual-isotope WBC/bone marrow scan, and FDG PET/CT. The final diagnosis of prosthetic joint infection (PJI) and/or loosening was based on the intraoperative findings and microbiological culture results and the clinical follow-up. Results: The diagnostic performance of dual-isotope WBC/bone marrow SPECT/CT for PJI showed a sensitivity of 100% (CI 0.74–1.00), a specificity of 97% (CI 0.82–1.00), and an accuracy of 98% (CI 0.88–1.00); for PET/CT, the sensitivity, specificity, and accuracy were 100% (CI 0.74–1.00), 71% (CI 0.56–0.90), and 79% (CI 0.68–0.93), respectively. Conclusions: In a standardized prospectively scheduled patient group, the results showed highly specific performance of combined dual-isotope WBC/bone marrow SPECT/CT in confirming chronic PJI. FDG PET/CT has an appropriate accuracy, but the utility of its use in the clinical diagnostic algorithm of suspected PJI needs further evidence.

## 1. Introduction

Total hip and knee arthroplasty (TKA and THA) surgeries are among the most clinically successful and cost-effective orthopedic procedures for end-stage degenerative, traumatic, and inflammatory joint disease. However, complications such as chronic pain, prosthetic joint infection or aseptic failure remain a challenge for health services, and timely identification and initiation of treatment are crucial. Prosthetic joint infection (PJI) can result in chronic pain, reduce function, and substantially decrease functional outcomes and morbidity. Chronic problems related to THA and TKA with negative laboratory, microbiology, and radiological results are challenging to diagnose and treat [1,2]. In the case of recurrent chronic painful prostheses, early differentiation between potential aseptic failure (AF), periprosthetic joint infection (PJI), or chronic pain is essential for choosing the respective treatment. In Scandinavian countries, the leading indication for revision surgery is AF followed by PJI, but as previously reported, patients undergoing revision surgery are at an 8 times higher risk of subsequent PJI than patients undergoing primary arthroplasty [2,3,4].

The diagnostic approach in patients with suspected PJI or AF is variable between countries and centers [5,6] and is usually guided by diagnostic criteria for PJI [5,7,8] and available imaging techniques. Advanced nuclear imaging techniques such as ^99m^Tc-HDP bone scintigraphy, radiolabeled leukocyte scintigraphy with or without combined computed tomography, or ^18^F-FDG PET/CT can be used to evaluate suspected PJI. However, the choice of the most accurate imaging procedure and optimal radiopharmaceutical remains controversial [9]. A literature search reveals different diagnostic accuracies using the same imaging techniques [5,10,11,12,13,14,15,16,17,18,19,20,21,22], with consecutive agreement that there is no single imaging modality to date (both in the field of radiology or nuclear medicine) that is able to diagnose all possible disorders with satisfactory accuracy [23]. Therefore, a combination of modalities remains necessary in many cases.

A recently published consensus document by Signore A. et al. [6] and Multidisciplinary Consensus Statements by Romano C.L. et al. [7] highlighted the current evidence-based diagnostic performances of different nuclear imaging techniques and proposed diagnostic flowcharts for PJI [6]. However, the final decision for an imaging technique remains highly dependent on local availability, time, cost, and expertise [7]. Moreover, the multidisciplinary approach is highly recommended.

The purpose of our study was to investigate the diagnostic value of advanced nuclear imaging for patients with chronic problems related to knee or hip prostheses in a prospective interdisciplinary study. The PRIS study was performed according to a structured multidisciplinary diagnostic algorithm incorporating advanced nuclear imaging, optimized sampling logistics, culturing methods, 16S rRNA gene polymerase chain reaction (PCR), and amplicon sequencing [2]. Our study included a subgroup of patients scheduled by the algorithm who underwent advanced nuclear imaging with ^99m^Tc-HDP SPECT/CT (bone scan), dual-isotope combined ^111^In-labeled WBC/^99m^Tc-Nanocoll bone marrow (dual WBC/bone marrow scan) SPECT/CT, and ^18^F-FDG PET/CT (FDG PET/CT). The aim of this study was to investigate the diagnostic accuracy of these imaging modalities to improve the diagnostics of patients experiencing problems after TKA or THA and help clinicians choose the most accurate diagnostic strategy and treatment.

## 2. Materials and Methods

**Figure A1:**
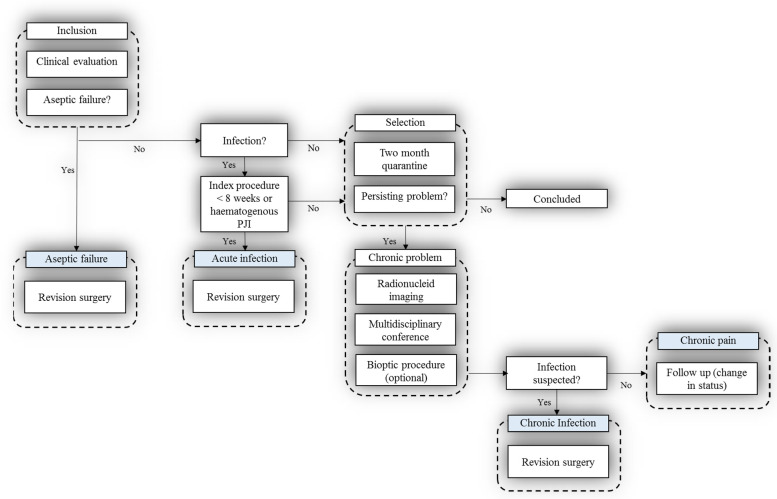
Diagnostic algorithm for the PRIS study.

This study was conducted in the North Denmark Region from December 2011 to January 2014 within the framework of an innovation consortium with the participation of clinical departments, universities, industry, and the Danish Technology Institute (Danish acronym PRIS). The Department of Orthopedic Surgery of Aalborg University Hospital was responsible for the inclusion, treatment, and coordination of the project. Patients presenting with a prosthetic problem related to either a TKA or THA were included and followed the multidisciplinary algorithm (Appendix A, Figure A1). The full project overview with inclusion criteria, detailed patient characteristics, pre- and postoperative findings, and a full description of the multidisciplinary algorithm was described by Khalid et al. [2,24].

### 2.1. Patient Population

A total of 156 patients with 163 arthroplasties were included in the PRIS project [2,24].

According to the project algorithm (Figure A1), fifty-five patients (26 women and 29 men, mean age 64 years) were included in a group for chronic problems (29 knees and 26 hips). They underwent extensive nuclear imaging on three consecutive days, including ^99m^Tc-HDP SPECT/CT (bone scan), dual-isotope combined ^111^In-labeled WBC/^99m^Tc-Nanocoll bone marrow (dual WBC/bone marrow scan) SPECT/CT, and ^18^F-FDG PET/CT (FDG PET/CT). The imaging results were evaluated at multidisciplinary conferences by specialists in nuclear imaging, orthopedic surgery, and clinical microbiology who guided further management. If findings were compatible with infection, a diagnostic procedure or revision surgery was recommended. In the absence of findings suggestive of PJI, patients were diagnosed as having a chronic pain problem, and follow-up for change in status was planned.

The basic patient demographics are shown in Table A1. The mean prosthesis age in our subgroup was approximately 3–4 years for both the hip and knee. There was no differentiation made regarding the type of implant.

### 2.2. Ethics

Approval of the PRIS project was obtained from the Research Ethics Committee for the North Denmark Region (N-20110022) and the Danish Data Protection Agency (2008-58-0028).

### 2.3. Reference Standard

The final diagnosis was obtained from microbiological data from perioperative samples or percutaneous biopsies and/or joint aspirates.

Sampling during revision surgery was identical regardless of indication. Before the administration of antibiotics, five periprosthetic synovial tissue biopsies were obtained, according to Kamme and Lindberg, as is the routine in the Department of Orthopedic Surgery. Intraoperative project samples followed a previously published protocol [25,26]. Project samples included triplicates of joint fluid, periprosthetic synovial tissue, and bone biopsies, and swabs from the surface of the prosthesis. This sampling strategy made it possible to evaluate experimental methods. Any removed prosthetic components were handled aseptically and subjected to sonication according to a previously published protocol [25]. Protocol samples were subject to bacteriological culturing for 14 days, 16S rRNA gene PCR followed by amplicon sequencing, and fluorescence in situ hybridization (FISH) (optional). Detailed microbiological sampling and culturing protocols and data from PRIS study were previously published by Larsen et al. [25,26].

**Figure A2:**
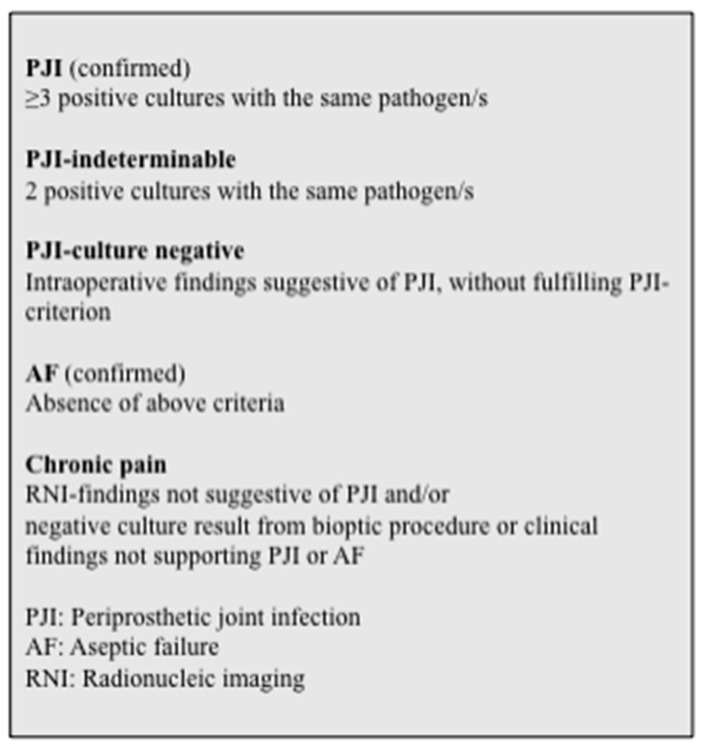
Definition of post-operative and post-radionuclide diagnosis.

In patients undergoing revision surgery, confirmation of PJI required positive culture reports for at least three of five periprosthetic soft tissue biopsies with identical microorganism(s). This criterion had been used by the Department of Orthopedic Surgery since the 1990s, and a validation study was previously performed for patients with knee arthroplasty. Less stringent criteria have been proposed by recent studies [27], and therefore, an additional analysis was performed for patients with two positive tissue biopsy cultures with identical microorganism(s). A diagnosis of culture-negative PJI was made if clinical findings, including intra-operative view, were suggestive of PJI without fulfilling other criteria for PJI [2,24] (Figure A2).

When invasive procedures were not available, patients were scheduled for clinical follow-up. The clinical follow-up data were evaluated by the post hoc multidisciplinary team (MDT) to classify these patients after the study ended. The median clinical follow-up period from imaging to final clinical MDT was 33–34 months (Table A1). The follow-up MDT team consisted of three consultants who were specialists in infectious disease, clinical microbiology, and orthopedic surgery. They were independently blinded to the patient identity and were asked to categorize each patient as (1) suspected of PJI, (2) PJI effectively ruled out, or (3) a conclusion was not possible. More details for clinical MDT are described by Khalid et al. [2].

Both the microbiological culturing results and the clinical follow-up results were considered the “gold standard” in this study.

### 2.4. Imaging Procedures

The nuclear imaging procedures were performed in the Department of Nuclear Imaging at Aalborg University Hospital from December 2011 to January 2014. The acquisition and imaging analysis were performed in accordance with the European Society of Nuclear Medicine (EANM) guidelines and the institutional procedures for all three imaging modalities. Both nuclear imaging and CT data were included in the image interpretation.

**Figure 1:**
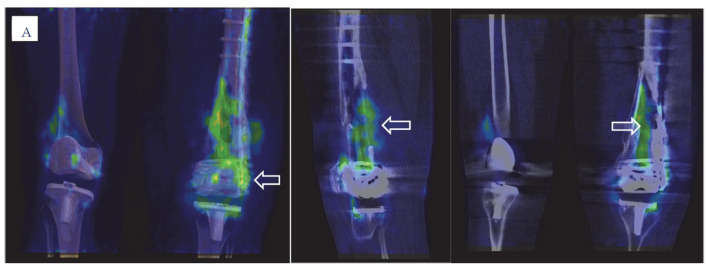

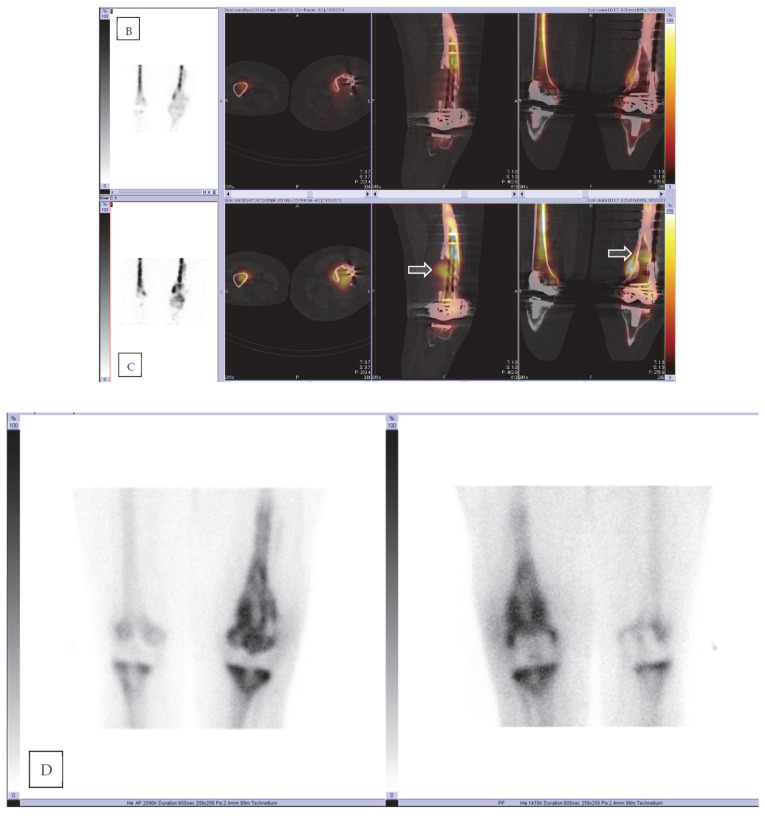

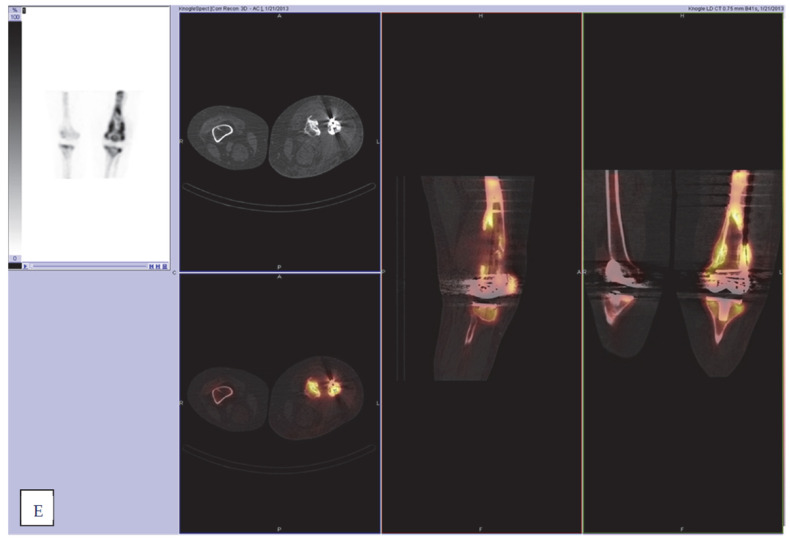
Three hybrid imaging modalities for the same patient with an infected knee prosthesis. (**A**) FDG PET/CT (showing high FDG uptake located in distal femoral bone marrow and around cortical bone defects, also around the screws in lateral femoral condyle (arrows)). (**B**,**C**) Dual-isotope combined In-labeled leucocyte SPECT/CT (**B**) and bone marrow SPECT/CT (**C**) (showing mis-matched focal leucocyte uptake (arrow) in distal femoral bone marrow and around cortical bone defects, also around the screws in lateral femoral condyle). (**D**,**E**) Bone scan; static uptake (**D**), SPECT/CT (**E**) (showing generally high uptake in bone tissue around the prosthesis and metal fixation both in distal femur and proximal tibia).

**Figure 2:**
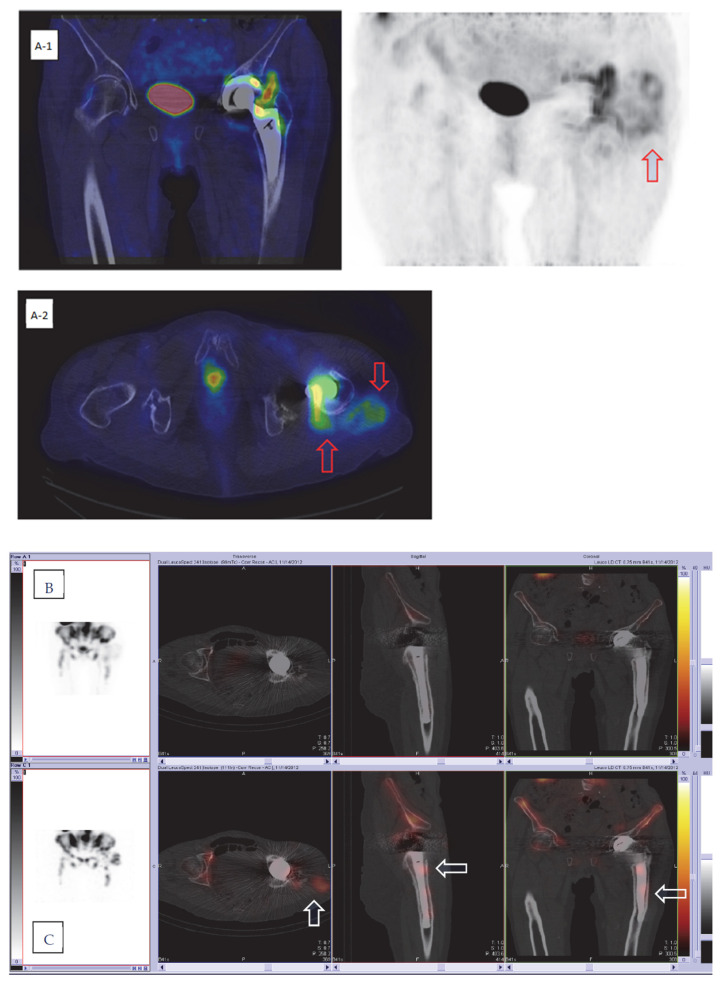

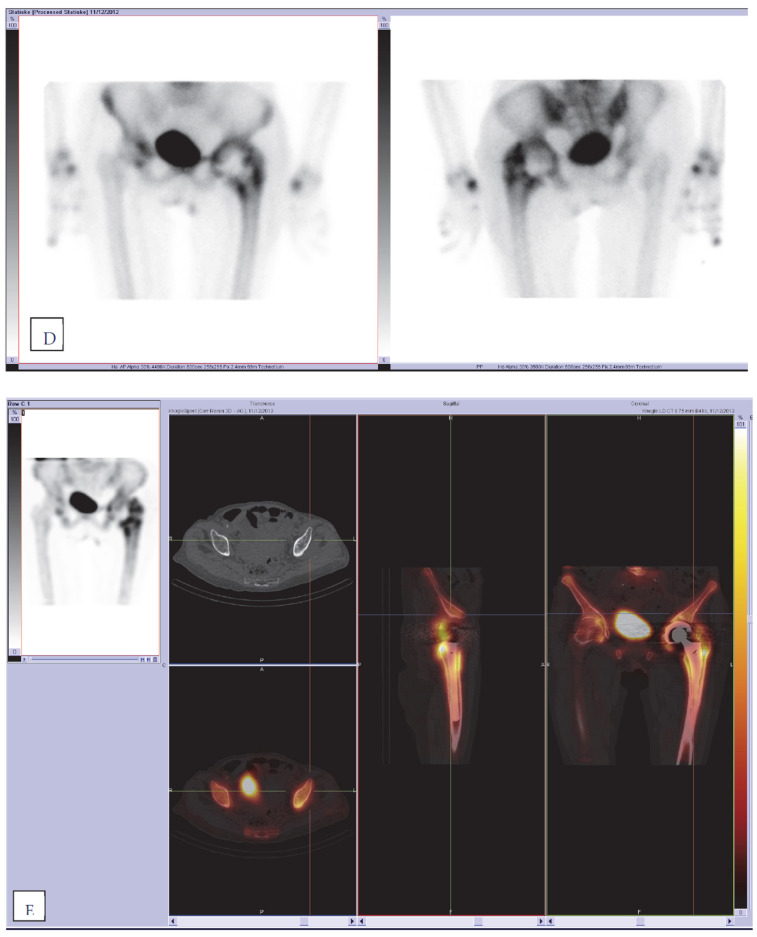
Three hybrid imaging modalities for the same patient with an infected hip prosthesis. (**A-1)** and (**A-2**) FDG PET/CT, showing high FDG uptake in the periprosthetic soft tissue communicating with intraarticular space (red arrows). (**B**,**C**) Combined In-labeled leucocyte SPECT/CT (**B**) and bone marrow SPECT/CT (**C**) (showing focal leucocyte uptake in the soft tissue (arrows) and mis-matched focal leucocyte uptake in the prosthesis bone interface in the femoral part (arrows). (**D**,**E**) Bone scan; static uptake (**D**), SPECT/CT (**E**) (showing generally high uptake in bone tissue around the prosthesis in proximal femur).

All 55 patients were scheduled for nuclear imaging investigation with ^99m^Tc-HDP SPECT/CT, combined ^111^In-labeled WBC/^99m^Tc-Nanocoll bone marrow SPECT/CT, and ^18^F-FDG PET/CT performed within a week (Figure 1 and Figure 2). The combined imaging results were reported and discussed at the multidisciplinary team (MDT) board in conjunction with the clinical data within a few days after radionuclide imaging. The MDT consensus was classified as positive for infection, negative for infection, or suspected of aseptic failure, and guided further management.

### 2.5. Acquisition Protocols

#### 2.5.1. Bone SPECT/CT

Bone scans with ^99m^Tc-HDP were performed as planar images followed by SPECT/CT with low-dose CT in accordance with the earlier European Society of Nuclear Medicine guidelines [28]. The images were acquired on a Siemens dual-head hybrid scanner (Symbia T16, Siemens Medical Solutions, Erlangen, Germany) with multipurpose, low-energy, high-resolution collimators. The mean injected activity of ^99^Tc-HDP was 750 MBq. Only late images approximately 2–3 h after tracer injection were acquired. Planar images were acquired over 10 min simultaneously in the anterior and posterior view, with 256 × 256 matrix and zoom factor 1.00. The study was then concluded by SPECT/CT acquisition with the following parameters: 20 s/view, 32 views, matrix size 128 × 128, zoom factor 1.00, flash 3D iterative reconstruction with scatter correction, and low-dose CT with 0.65–5.0 mm slice thickness.

#### 2.5.2. Dual-Isotope Combined ^111^In-Labeled Leucocyte/^99m^Tc-Nanocoll Bone Marrow SPECT/CT

The white blood cell (WBC) labeling procedure with ^111^In was performed in accordance with the European Society of Nuclear Medicine guidelines for the labeling of leucocytes with ^111^In-oxine [29]. The labeling efficiency was >85%. Planar imaging of the lungs was performed 30 min after reinjection of labeled leucocytes for quality control of the radionuclide labeling procedure. The mean injected activity of ^111^In-labeled leucocytes was 20 MBq. On the next day, the patient was injected with ^99m^Tc-Nanocoll with an activity of 500 MBq for bone marrow imaging. Simultaneous dual-isotope scanning combining ^111^In-labeled WBC scintigraphy with ^99^Tc-Nanocoll bone marrow scintigraphy with SPECT/CT (low-dose CT) was performed 24 h after reinjection of ^111^In-labeled leucocytes and 45–60 min after injection of ^99m^Tc-Nanocoll. The acquisition was performed on a Siemens Hybrid scanner (Symbia T16, Siemens Medical Solutions, Erlangen, Germany) in compliance with the European Society of Nuclear Medicine guidelines [30].

Uptake parameters were as follows: static uptake 15 min, matrix size 256 × 256, zoom factor 1.00 followed by dual-isotope SPECT/CT 45 sec/view, 32 views, matrix size 128 × 128, zoom factor 1.00, iterative reconstruction, collimator MELP (Medium-Energy Low Penetration).

#### 2.5.3. ^18^F-FDG PET/CT

^18^F-FDG PET/CT scans were acquired on a GE Discovery VCT PET/CT scanner (GE Healthcare, Waukesha, WI, USA) in accordance with EANM procedure guidelines for tumor imaging [31] and EANM/SNMMI guidelines for ^18^F-FDG use in inflammation and infection [32]. Patient preparation with at least 4 or 6 h of fasting prior to the administration of ^18^F-FDG, blood glucose levels lower than 11 mmol/l, and a resting time of approximately 60 min after tracer injection were obligatory. The mean injected activity of ^18^F FDG was 370 MBq. The CT portion was acquired with a low dose and 0.65–5.0 mm slice thickness. Corrected and uncorrected transaxial, sagittal, and coronal images were acquired with an iterative reconstruction algorithm and an existing metal artifact reduction algorithm.

### 2.6. Image Interpretation and Diagnostic Classification

At least two board-certified nuclear physicians, one junior and one senior, and one board-certified radiologist working at the nuclear medicine department evaluated all three hybrid imaging examinations. Each imaging modality was reported as in daily clinical practice, independently from the reviewers, and was blinded regarding the results of other imaging studies and clinical or biochemical data.

**Figure 3:**
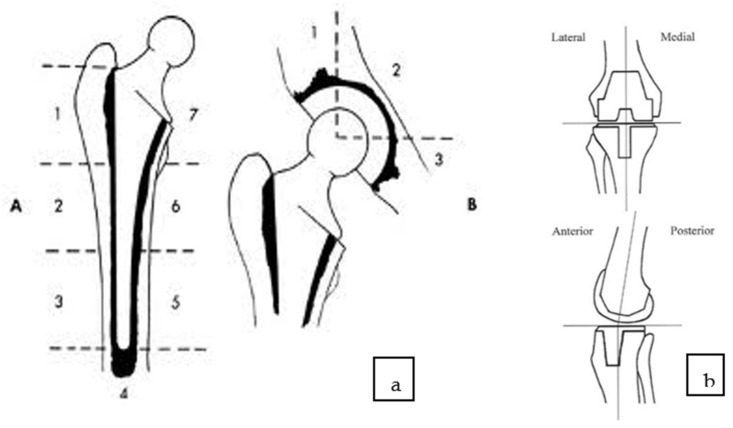
The uptake on images was registered according to the Gruens zones for femoral component (1–7) (A) and Charnley deLee (B) zones for acetabular component (1–3) for hip prostheses (**a**) and a custom-made scheme for knee prostheses (**b**).

The evaluation criteria for FDG PET/CT were based on the pattern and location of FDG uptake. Increased tracer uptake was reported if localized at the bone–prosthesis interface, in the periprosthetic soft tissue, related to the capsule and/or outside the joint, and in the regional lymph nodes. The standardized system to localize the uptake at the bone–prosthesis interface was used in all three modalities: Charnely DeLee for the acetabulum, Gruen zones for the femoral implant, and the standardized localization scheme for the knee (Figure 3). The background SUV values were calculated in the VOIs located in the region of gluteus/hip muscles, as standard values in the lever or aorta were not possible due to not being acquired. The criteria we used to differentiate nonspecific FDG uptake from patterns suspected of infection/loosening were described by Reinartz P. et al. [33] Mumme et al. [34], Gemmel F. et al. [18], and Manthey et al. [35].

The evaluation criteria for dual-isotope WBC/bone marrow imaging were based on pathological mismatch uptake around the prosthesis (focal uptake on WBC scan with no uptake on bone marrow scan) or leucocyte uptake in the surrounding soft tissue or regional lymph nodes [36,37,38,39,40] (Figure 1 and Figure 2).

In addition to the clinical rapport, each nuclear medicine physician was asked independently to conclude whether tracer uptake patterns on dual-isotope and PET/CT imaging were (a) suspected for PJI (1) or (b) not suspected for PJI (0). If the results were not agreed upon or were equivocal, they were additionally discussed and reported as final imaging consensus. Discussion on the imaging results was mostly needed on the reporting the FDG PET/CT images and only few on the dual-isotope scans. The detailed interobserver agreement analysis was not performed in this study.

Bone SPECT/CT results were reported as in daily clinical practice without classification for suspected PJI but were highly valuable for demonstrating the normal appearance and various post-surgical complications.

The results of all three hybrid imaging examinations were ultimately discussed at the post-imaging MDT conferences in conjunction with the clinical and biochemical data. The remaining few equivocal imaging results were classified as suspected of infection/aseptic loosening or negative: one case on the dual-isotope imaging and six cases on FDG PET/CT. As previously stated, the MDT consensus guided patent management.

### 2.7. Statistics

Decisions on infection based on dual-isotope or FDG PET/CT images were compared to decisions made by the clinical follow-up MDT. Sensitivities, specificities, positive and negative predictive values, and overall accuracy were computed, with microbiology and clinical MDT decisions considered the gold standard. For the estimates, 95% confidence intervals were calculated for binomial proportions using the exact method.

All analyses were performed in R version 3.6.3 [41].

## 3. Results

### 3.1. Patients

**Figure 4:**
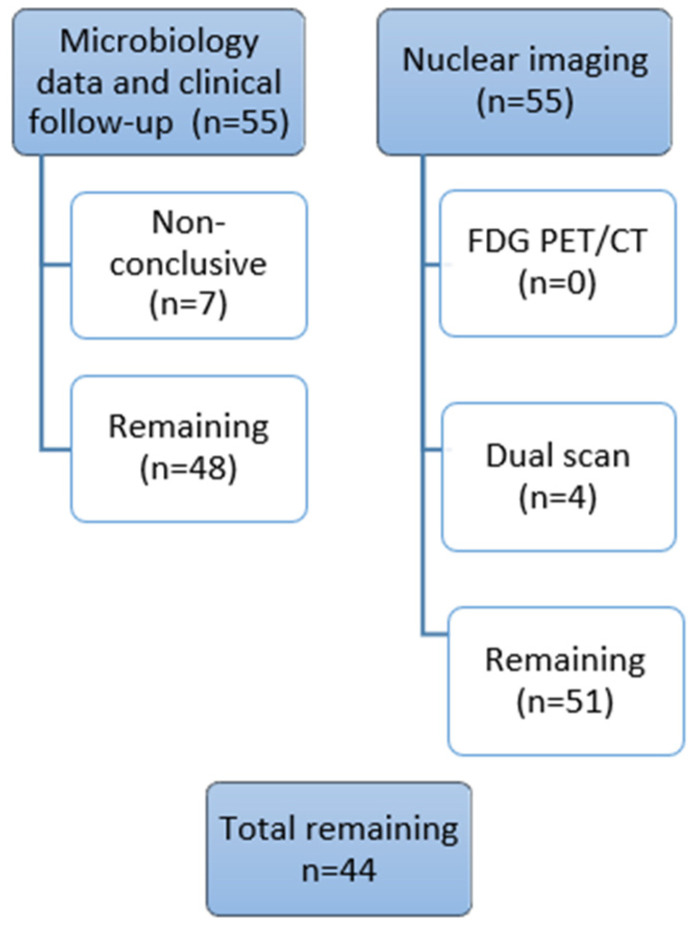
Patients excluded from the statistical analysis.

Twenty-three patients (42%) (with 12 hip and 11 knee prostheses) underwent percutaneous biopsies/joint aspirates or revision surgery after imaging, and the final diagnosis was obtained by microbiological culturing. Twenty patients (38%) were scheduled for clinical follow-up due to negative results on all three scans in six patients and in 14 patients with no suggestive periprosthetic infection or loosening on both FDG PET/CT and dual-isotope SPECT/CT. The remaining patients either missing dual-isotope scans and/or patients with findings suggestive of PJI or aseptic loosening who did not undergo surgery due to other reasons (severe comorbidity, mild symptoms, etc.) were scheduled for clinical follow-up or underwent revision surgery later on beyond the study for PJI [2]. Clinical follow-up (median follow-up 33–34 months) with the final post hoc MDT evaluation was performed on all 55 patients. Patients excluded from the statistical analysis are shown in Figure 4.

### 3.2. Imaging and Clinical Follow-Up

The results on all three hybrid imaging modalities according to the classification for suspected PJI/loosening are presented in Table 1.

As previously indicated, isotope distribution patterns and morphological changes on bone SPECT/CT imaging were reported according to clinical practice as part of diagnostic imaging tests describing tracer uptake for suspected loosening and/or infection but without direct classification of possible PJI. Additionally, other abnormalities, post-surgical complications, and degenerative changes were reported on bone SPECT/CT, such as heterotopic ossification (in seven patients), uptake in the patella with or without signs of degeneration (in nine patients), fluid collection (in two patients), endosteal scalloping (in three patients), and fracture in the acetabulum with a changed position of the acetabular cup (in one patient).

PJI for both the knee and hip was suspected in 17 patients (33%) on dual-isotope imaging and in 27 patients (49%) on FDG PET/CT scanning, with false positives in 10 patients (21%) on PET/CT (Table 2).

The estimates shown in Table 2 on diagnostic accuracy are extremely high, and the confidence intervals are skewed to the left. The high estimates of sensitivity and specificity can be due to randomness in small samples (*n* = 44, 48), since each participant represents several percentage points. Hence, confidence interval midpoints are chosen for expected values of sensitivity and specificity (except for FDG PET/CT specificity) and summarized in Table 3.

We conducted additional analysis to evaluate the inconclusive decisions from the clinical follow-up MDT. The equivocal MDT results were “converted” to values opposite to decisions based on FDG PET/CT and/or dual-isotope scans, representing the so-called “worst-case” scenario (Table 4), and the diagnostic performance is presented in Table 5.

## 4. Discussion

Periprosthetic joint infection is the most serious complication after hip or knee replacement and may lead to repeated surgical interventions, prolonged hospitalization, and very high costs [6]. Late, chronic, low-grade infection is mostly associated with nonspecific symptoms and remains a challenging diagnostic problem.

In this prospective PRIS study, 156 patients representing 163 cases of TKA or THA were recruited and assessed by the use of a multidisciplinary diagnostic algorithm including multimodal nuclear imaging (on the subgroup of 55 patients) and extended microbiological diagnostics with optimized sampling logistics, culturing methods, 16S rRNA gene polymerase chain reaction (PCR), and amplicon sequencing [2]. To our knowledge, this is the first study of its kind [2]. The hypothesis was that diagnosis in patients experiencing post-hip or -knee replacement problems can be improved by following a structured diagnostic multidisciplinary algorithm.

The study was prospectively designed and involved a highly specialized multidisciplinary team throughout the project. It is well known that healthcare today has an increasing need for a team-based approach to care requiring interdisciplinary collaboration [42]. Cooperation and effective communication between the entire multidisciplinary team can maximize patient outcomes. Chronic PJI can only be cured by prosthesis replacement performed as a one- or two-stage procedure with concomitant antibiotic therapy [43] but should be applied when relevant due to the high risk of secondary infection. The results of PRIS study showed that surgical revision was obviated in approximately 20% of patients after using a strict diagnostic workup [2]. The strength of the study was the prospective design and multidisciplinary cooperation, serving as tools in personalized patient treatment.

In this analysis of the patient subgroup, we report the diagnostic value of advanced hybrid nuclear imaging in patients with chronic problems after total hip or knee arthroplasty. It is important to emphasize that nuclear imaging significantly impacted clinical decisions on patient management in this prospective study. Imaging data from all imaging modalities were discussed together with clinical and biochemical patient data among a multidisciplinary team before further invasive procedures were performed. The purpose of our study was to investigate the diagnostic value of advanced hybrid imaging to include the best technique in future clinical diagnostic workups for diagnosing PJI. Bone scans, including three-phase bone scintigraphy, are the most widely used screening modality for the diagnosis of PJI [6], as they are very sensitive to any bone remodeling. This examination should be avoided in the first years after surgery [44], as physiological bone remodeling probably takes place in the first years after joint replacement surgery and is also dependent on the type of prosthesis [6,45]. When performing bone scans with SPECT/CT for PJI, one should remember that the most important contribution of the modality is its very high negative predictive value (NPV) [6,7,22,23]. If a bone scan is negative, an additional WBC scan can be avoided with a sensitivity of 80% and a specificity of 99.5% [46,47,48] and is considered strong evidence against the presence of an infection [6,16,49]. Therefore, normal physiological tracer uptake in symptomatic patients is a good indicator for an alternative cause of pain rather than post-operative complications [50]. In our study, the normal tracer distribution on bone SPECT/CT was demonstrated in 6 out of 55 patients, which was confirmed by following dual-isotope SPECT/CT and FDG PET/CT. The diagnosis of periprosthetic joint infection and/or AF was excluded in these patients, and they were referred to the pain clinic for further investigations and were preserved from undergoing unnecessary revision operations.

Conversely, the specificity of bone SPECT/CT alone reported in the literature is usually lower than sensitivity, especially for PJI. The evidence in the literature shows heterogeneity of data concerning the diagnostic accuracy of bone scintigraphy but demonstrates lower specificity than all other nuclear imaging modalities [51]. Several meta-analyses showed a pooled specificity of 56% (95% CI, 47–64%) for bone scintigraphy [51] or a pooled sensitivity and specificity of 80% (95% CI, 72–86%) and 69% (95% CI, 91–99%) [9]. The reported isotope distribution patterns on bone scans are less specific for infection and a bone scan is not recommended for PJI diagnosis as a single modality. On the contrary, a recent study published by Bäcker et al. showed very high sensitivity, specificity, positive predictive value, and negative predictive value for loosening of SPECT/CT, of 93%, 97%, 90%, and 100%, respectively [52]. The authors reported the results of MRI and dual-phase bone scan with SPECT/CT with very high accuracy of both modalities. Loosening in this study was diagnosed according to the combined SPECT/CT criteria published by Dobrindt et al. [53].

It should be additionally mentioned that bone scintigraphy, especially in combination with CT, can help to assess other abnormalities causing chronic pain, such as heterotopic ossification, “hot patella” sign, periprosthetic fractures, wear-associated osteolysis, histiocytic reaction, or fluid collections [52,54].

In our institution, bone SPECT/CT remains the first nuclear imaging modality in patients with a low probability of infection in THA and TKA, which is in agreement with recently suggested diagnostic flowcharts [6,22]. The results of bone SPECT/CT in our study were not categorized as possible PJI, as it was beyond the scope of this study and therefore not included in the statistical analysis. The aim of performing this modality was to detect possible periprosthetic loosening as much as negative cases or other possible post-operative complications causing chronic pain and comparing findings with dual-isotope SPECT/CT and FDG PET/CT.

In the case of a positive bone scan, another nuclear imaging modality is necessary in patients suspected of having PJI. The standard of care in these cases is radiolabeled WBC scintigraphy [9,22,49], with ^99m^Tc_HMPAO or ^111^In-oxine, which can confirm or rule out infection with high diagnostic accuracy, especially if performed using EANM standardized criteria for the labeling procedure, acquisition, and interpretation [9,11,40,51]. Radiolabeled leucocytes accumulate not only in infections, but also physiologically in the active reticuloendothelial component of bone marrow. The distribution of bone marrow is affected by joint prostheses, making it difficult to differentiate labeled leukocyte accumulation in infection from accumulation in atypically located but otherwise normal marrow. This differentiation is accomplished by complementary bone marrow imaging with ^99m^Tc-sulfur colloid. Leukocytes and sulfur colloids both accumulate in the reticuloendothelial cells of the bone marrow, and tracer uptake on both scan types indicates the presence of physiological uptake in atypically located bone marrow and thus allows us to reduce the number of false-positive cases in WBC scintigraphy.

In vitro leucocyte labeling with ^111^In-oxine has been used in humans for infection imaging for more than 50 years [55], and since 1988 has been largely replaced by ^99m^Tc-HMPAO [56]. Nowadays, WBC labeling with ^111^In-oxine or ^99m^Tc-HMPAO is a well-established technique in Europe [29,56], but the procedure requires highly specific laboratories and qualified personnel validated for the WBC labeling process and handling the blood components of the patient who could potentially be infected, and it is time consuming. WBC scans require 24 to 72 h before results are obtained. Despite these disadvantages, scintigraphy with labeled autologous WBSs is a widely used method and remains the most specific imaging technique for detecting sites of infection. The study by Palestro et al. from 1990 demonstrated high diagnostic accuracy on combined ^111^In-labeled leucocyte and bone marrow imaging in PJI; the results demonstrated a sensitivity, a specificity, and an accuracy of 100%, 97%, and 98%, respectively [37]. Some later published papers reported slightly different diagnostic accuracy for combining techniques, ranging from 86% to 98% [18]; the sensitivity, specificity, and accuracy reported by Love C et al. were 100%, 91%, and 95%, respectively [57,58]; those reported by El Esper et al. were 80%, 94%, and 91%, respectively [59]; and those reported by Brammen L et al. were 60%, 97%, and 90%, respectively [60]. Different results in reported studies can be explained by the use of different acquisition protocols and interpretations. The low sensitivity reported by Brammen et al. could be rated to 100% after reviewing imaging by one of the leading nuclear medicine specialists, demonstrating the importance of standardized image interpretation by experts [60]. The latest reviews and meta-analysis obtained from papers with consistent combined techniques show a pooled specificity of 92% (95% CI, 84–97%) [51] and diagnostic accuracy ranging from 83% to 98% for both hip and knee prosthesis infections [49]. In our study, we performed a combined dual-isotope ^111^In-labeled WBC/^99m^Tc-Nanocoll bone marrow scan with the SPECT/CT technique, which was standardized in accordance with the European Society of Nuclear Medicine guidelines. This advanced hybrid combination of two nuclear imaging modalities with CT has been shown to have the highest diagnostic accuracy. Adding SPECT/CT can increase the diagnostic accuracy in the case of PJI due to its better resolution and morphological information [6,54,61]. The specificity for SPECT/CT compared with SPECT alone combined with different tracers can be increased by up to 38% [6].

The results of dual-isotope imaging SPECT/CT in our study demonstrate high sensitivity, specificity, diagnostic accuracy, and NPV values, with 100%, 97%, 98%, and 100%, respectively, compared to the gold standard and comparable to the overall accuracy reported in the literature. However, there are some limitations which must be discussed. The estimates on diagnostic accuracy presented extremely high, and the confidence intervals skew to the left. When corrected to skewness in confidence intervals, the sensitivity and specificity are 88% and 92%, respectively. Another limitation of this analysis is that seven patients lacking the gold standard were excluded due to equivocal clinical follow-up results. When these inconclusive clinical results were categorized in contrast to imaging results, the sensitivity, specificity, and diagnostic accuracy were significantly lower at 76%, 88%, and 84%, respectively. Interestingly, the imaging results for all seven patients were consistent on both dual-isotope and PET/CT; therefore, the accuracy in the “worst-case” analysis may be considered underestimated.

In our institution, dual-isotope WBC/bone marrow imaging performed with the SPECT/CT technique is included in the diagnostic imaging algorithm in daily clinical practice for THA and TKA chronic periprosthetic problems if bone scans cannot rule out PJI. The labeling in our institution is performed with ^111^In-oxine. When comparing imaging with ^99m^Tc-HMPAO to ^111^In-oxine-labeled WBC, there are some advantages and disadvantages. The main advantage of ^111^In-oxine over ^99m^Tc-HMPAO is the higher labeling efficiency (LE) and less efflux of radioactivity from the labeled WBC. The most important disadvantage, however, is the radiation exposure of labeled cells, critical organs (spleen), and the whole body to ^111^In-oxine, which is substantially higher than that from ^99m^Tc-HMPAO. Planar images obtained with ^111^In-labeled WBC are of substantially lower quality than those obtained with ^99m^Tc-labeled WBC. SPECT images of ^111^In-labeled WBC are of very low quality as well, unless the acquisition time is largely increased. On the other hand, the use of ^111^In-oxine-labeled WBC does not interfere with imaging of ^99m^Tc-nanocolloids, because different energy windows can be used to detect ^99m^Tc and ^111^In simultaneously [29,56]. Fluorine-18 fluorodeoxyglucose positron emission tomography (^18^F-FDG-PET), which is primarily used for the localization of malignancy, has also demonstrated utility for the detection of infection or inflammation. FDG is a glucose analog that is primarily taken up by high-glucose-consuming cells, including inflammatory cells such as neutrophils and monocytes [62]. The nonspecific mechanism of ^18^F-FDG uptake has been shown to be beneficial in terms of its high sensitivity and high negative predictive value but has a limitation of low specificity for imaging inflammation and infection. The diagnostic performance of ^18^F-FDG PET/CT in detecting PJI in hip and knee replacements has been increasingly proven in the literature to not be inferior to labeled leukocyte scintigraphy [1,5,9,49,51]. The joint EANM/SNMMI guidelines for the use of FDG in inflammation and infection reported an overall sensitivity of 96% for FDG PET and a specificity of 98% for knee and hip PJI [32]. A recent meta-analysis of the diagnostic performance of FDG PET/CT reported a poled sensitivity of 86% (95% CI, 80–90%) and a pooled specificity of 93% (95% CI, 90–95%) for hip prostheses for FDG PET/CT [9]. Another meta-analysis of the diagnostic performance of FDG PET/CT reported a pooled sensitivity of 70% (95% CI, 56–81%) and a pooled specificity of 84% (95% CI, 76–90%) for knee prostheses [51]. Results from a recently published meta-analysis by Kim showed a pooled sensitivity of 0.88 (95% CI; 0.80–0.93) and a pooled specificity of 0.89 (95% CI; 2 0.83–0.93) for the detection of PPI of lower limb arthroplasty of the 19 included studies [63].

There are few published papers that directly compare FDG PET/CT and WBC scintigraphy in prosthetic infections, and the tested interpretation criteria are different [6]. The results from these studies are heterogeneous [5], but all papers confirm the lower specificity of FDG PET/CT compared with WBC scintigraphy in PJI [57,58,64,65,66]. In our study, the overall accuracy was significantly lower for FDG PET/CT (79%) than for dual-isotope WBC/bone marrow scintigraphy (98%). In the case of the “worst-case scenario”, the diagnostic accuracy for FDG PET/CT was lower compared with dual-isotope WBC/bone marrow scintigraphy. The results from our study are comparable to the accuracy reported in the literature, but the heterogeneity of the reported diagnostic performance of FDG PET/CT in PJI in the literature should be mentioned. Even though the diagnostic accuracy of FDG PETC/CT in pooled data in meta-analysis was high, the ranges for both sensitivity and specificity in individual studies were quite large (sensitivity 28–91% and specificity 34–97%) [6]. As reported by Signore et al., this is largely attributable to the differences in study design and interpretation criteria [6]. Therefore, standardization of acquisition protocols, diagnostic criteria, and reference standards is required for further validation of the method [5,67]. Furthermore, ^18^F-FDG PET/CT has been increasingly used in evaluating not only in oncological cases, but also patients with different infectious and inflammatory diseases, fever of unknown origin, as well as spondylodiscitis [7], in which interpretation of periprosthetic FDG uptake can be necessary due to the increasing number of joint replacements in the population. Thus, validated uptake interpretation criteria are necessary.

In our study, FDG PET/CT imaging was reported as suspected for PJI in 27 patients, where 10 cases (21%) (six knee and four hip prostheses) reported false positivity compared to the gold standard. In this false-positive group, three patients did not undergo dual-isotope scans due to technical issues, but seven patients underwent either revision (six) or joint aspiration with negative microbiological culturing results. In this group, uptake on FDG PET/CT was suspected due to more intense uptake in either periarticular soft tissue and/or the bone–prosthesis interface. In one case (TKA), both dual-isotope and FDG PET/CT were reported as suspected for PJI, where increased FDG and leucocyte uptake was reported in periarticular soft tissue and capsule and most likely represented tissue-reactive changes and/or synovitis. The limitations of the study should be discussed. One of the limitations of the study was that bioptic procedures and microbiological culturing were not feasible for a sizable portion of the patients. Therefore, both microbiological culturing and clinical follow-up results served as the gold standard in our study. Joint aspiration was discouraged in the initial evaluation due to possible interference with nuclear imaging. After imaging, joint aspiration or biopsies were discouraged for 38% of the patients due to negative imaging results. Joint aspiration itself involves a risk of infection, and its sensitivity is highly variable [6]. False-negative cultures are reported in the literature in approximately 20% of PJIs [7]. In our study, in five patients (11%), the invasive procedures were not performed despite both dual-isotope and ^18^F-FDG PET/CT imaging being suspected for PJI and the procedures were recommended. These patients did not undergo surgery due to severe comorbidities, mild symptoms, and other factors. To overcome this limitation, all patients were clinically followed up with and ultimately evaluated at the clinical follow-up and post hoc MDT. A further limitation is previously mentioned few equivocal imaging studies in which consensus between the readers could not be achieved and the results were discussed at the post-imaging MDT together with clinical data. The study was prospectively designed, addressed the daily clinical situation, and, in some cases, imaging results can be best interpreted and used in conjunction with the patient’s specific presentation [68]. The rest of the imaging results were not changed after the multidisciplinary team conferences.

A further limitation of the study was that seven patients were excluded from the final statistical analysis due to inconclusive final clinical MDT decisions. Interestingly, the results for both dual-isotope WBC/bone marrow scintigraphy and ^18^F-FDG PET/CT were consistent in all seven patients (Table 3), but no consensus could be reached from the clinical point of view at the time of the study. This limitation was fulfilled by additional statistical analysis, which we previously discussed.

One of the major limitations of the study is the small sample size. Post hoc power analysis [69,70] with the shown sensitivity and specificity in our study and CI 0.1 showed extremely high sample sizes for both imaging modalities (around 13,000 for knee PJI and 3500 for hip) calculated for the incidence of PJI in the cohort [71]. Therefore, the superiority of one imaging modality should be determined with caution.

Exposure to ionizing radiation is additionally an important issue. The level of radiation exposure of nuclear medicine examinations is similar to that of CT scans, ranging from 2 to 15 milliSieverts (mSv), with the highest radiation exposure for tumor imaging with FDG PET/CT [10]. In our study, an individual risk assessment was performed prior to every examination, with patients’ acceptance. However, three different nuclear medicine imaging modalities are not recommended in a daily routinely clinical practice for PJI in THA and TKA, as both WBC/bone marrow scan and FDG PET/CT show high diagnostic accuracy and can be selected according to local rules, expertise, and technical availabilities. Diagnostic strategies, though, differ between countries and nuclear imaging can be less important and less used than clinical examination and aspiration for further decision making.

## 5. Conclusions

We found a high diagnostic accuracy of both dual-isotope WBC/bone marrow SPECT/CT and FDG PET/CT for chronic PJI in THA and TKA. In accordance with published papers, our study confirms an accuracy for combined WBC/bone marrow imaging of >90%, indicating that it is the most specific imaging technique and the imaging modality of choice for diagnosing PJI. The specificity and overall accuracy of FDG PET/CT remain lower than those of combined WBC/bone marrow scintigraphy.

The lower specificity and heterogeneity of published diagnostic accuracy of FDG PET/CT emphasize the importance of standardization of both the reconstruction parameters and the interpretation criteria. Therefore, standardization and validation of this method for PJI are needed in larger studies.

Overall, our study shows that advanced nuclear imaging can serve as an important tool in the diagnostic work-up of PJI, both as a “rule out” and a “rule in” test. However, equivocal cases are most challenging, and the final decision remains dependent on a combination of clinical, laboratory, microbiology, and imaging tests. Therefore, interdisciplinary cooperation can optimize diagnostics and enable personalized patient treatment.

## Figures and Tables

**Table 1:** Results of the hybrid imaging.

Imaging Modality	No Increased Uptake		Increased Uptake				Total Number
			PJI Suspected		PJI Not Suspected		
	THA	TKA	THA	TKA	THA	TKA	
Bone SPECT/CT	4	2	*	*	*	*	55
Dual-isotope SPECT/CT	14	17	8	9	0	3	51
FDG PET/CT	8	5	13	14	4	11	55

* Not classified according the PJI.

**Table 2:** Diagnostic performance of dual-isotope SPECT/CT and FDG PET/CT when excluding patients with equivocal clinical follow-up results. TP, true positive; FP, false positive; TN, true negative; FN false negative. CI, confidence interval; *n*, number of performed tests; PPV, positive predicted value; NPV, negative predictive value.

Imaging Modality	Dual-Isotope (*n* = 44)	FDG PET/CT (*n* = 48)
TP	13	14
FP	1	10
TN	30	24
FN	0	0
Sensitivity [95% CI]	1.00 [0.75;1.00]	1.00 [0.77;1.00]
Specificity [95% CI]	0.97 [0.83;1.00]	0.71 [0.53;0.85]
PPV [95% CI]	0.93 [0.66;1.00]	0.58 [0.45;0.70]
NPV [95% CI]	1.00 [0.88;1.00]	1.00 [0.86;1.00]
Accuracy [95% CI]	0.98 [0.88;0.99]	0.79 [0.65;0.90]

**Table 3:** Diagnostic performance of dual-isotope SPECT/CT and FDG PET/CT when correcting confidence intervals.

Imaging Modality	Sensitivity	Specificity
Dual-isotope	0.88	0.92
FDG PET/CT	0.89	0.71

**Table 4:** Equivocal MDT results categorized to the opposite decision among decisions based on dual-isotope and FDG PET/CT imaging as the “worst-case scenario”. 1, PJI suspected; 0, PJI not suspected; 2, equivocal result.

Patient Nr.	Follow-Up MDT Results		FDG PET/CT Results	Dual-Isotope Results
	Primary	“Worst-Case”		
33	2	1	0	0
49	2	1	0	0
9	2	1	0	0
12	2	1	0	0
11	2	0	1	1
1	2	0	1	1
21	2	0	1	1

**Table 5:** Diagnostic performance of dual-isotope and FDG PET/CT in the “worst-case scenario” with equivocal MDT results categorized to the opposite decision among decisions based on dual-isotope and FDG PET/CT scans. TP, true positive; FP, false positive; TN, true negative; FN, false negative. CI, confidence interval; *n*, number of performed tests; PPV, positive predicted value; NPV, negative predictive value.

Imaging Modality	Dual-Isotope (*n* = 51)	FDG PET/CT (*n* = 55)
TP	13	14
FP	4	13
TN	30	24
FN	4	4
Sensitivity [95% CI]	0.76 [0.50;0.93]	0.78 [0.52;0.94]
Specificity [95% CI]	0.88 [0.73;0.97]	0.65 [0.47;0.80]
PPV [95% CI]	0.76 [0.50;0.93]	0.52 [0.32;0.71]
NPV [95% CI]	0.88 [0.73;0.97]	0.86 [0.67;0.96]

## Appendix A

**Table A1:** Patient demographics.

Basic Demographics	Hip	Knee
Patient age, years (mean, SD)	64.2 (14.4)	64.4 (12.2)
Gender, number		
Males (*n*)	16	13
Females (*n*)	9	17
Joint (*n*)	26	29
Prosthesis age, months (median, range)	38 (4–120)	41.5 (8–176)
Surgical procedures		
Revision (*n*)	10	9
Joint aspiration (*n*)	2	1
Follow-up period from imaging to final clinical MDT, months (median, range)	33 (6–45)	34 (21–42)
